# Clinical Course of Bacillus Calmette-Guerin Lymphadenitis

**DOI:** 10.3390/children9050610

**Published:** 2022-04-25

**Authors:** Dayoung Ko, Ji-Won Han, Joongkee Youn, Hee-Beom Yang, Chaeyoun Oh, Ki-Wook Yun, Hyun-Young Kim

**Affiliations:** 1 Department of Pediatric Surgery, Seoul National University Children’s Hospital, Seoul 03080, Korea; kodayoung@gmail.com (D.K.); hancci79@gmail.com (J.-W.H.); jkyoun@gmail.com (J.Y.); 2 Department of Surgery, Seoul National University Bundang Hospital, Seongnam 13620, Korea; eeulere@naver.com; 3 Department of Surgery, Korea University Ansan Hospital, Ansan 15355, Korea; chaeyoun.o@gmail.com; 4 Department of Pediatrics, Seoul National University Children’s Hospital, Seoul 03080, Korea; pedwilly@gmail.com; 5 Department of Pediatric Surgery, Seoul National University College of Medicine, Seoul 03080, Korea

**Keywords:** child, lymphadenitis, *Mycobacterium bovis*, vaccination

## Abstract

Bacillus Calmette-Guerin (BCG) vaccination can cause lymphadenitis. The purpose of the current study was to describe patient characteristics and clinical courses of lymphadenitis associated with BCG vaccination. A total of 171 patients who visited a tertiary hospital with a diagnosis of BCG-associated lymphadenitis between January 2012 and June 2017 were included. The diagnostic criteria were a history of BCG vaccination on the symptomatic side, absence of tenderness and raised temperature over the swelling, absence of fever and constitutional symptoms, and isolated axillary (or supraclavicular/cervical) lymph node (LN) enlargement. Treatment strategies included observation, antibiotics, incision and drainage or needle aspiration (I&D/NA), and surgical excision. The median follow-up period was 40 days (range 1–1245 days). The median age at the first visit was 5.5 months (range 0.9–83.7 months). The most common location was the axilla (81.3%). The respective numbers of patients managed via observation, I&D/NA, antibiotics, and surgical excision were 99, 47, 5, and 20. LNs were significantly more enlarged in the I&D/NA group than in the antibiotics group and the observation group. The respective times taken for residual lesions to reduce to < 20% were approximately 3 months, 4 months, and 5 months in the antibiotics, observation, and I&D/NA groups. The surgery group had significantly fewer residual lesions than the observation group at the last visit, but there was no significant difference in current residual lesions between the groups. LNs were significantly larger in the I&D/NA group. The surgery group exhibited the least residual lesions at the last visit, but there was no significant difference in current status.

## 1. Introduction

Bacillus Calmette–Guerin (BCG) is a live attenuated vaccine that was developed in 1921 to prevent tuberculosis caused by *Mycobacterium bovis* [1,2]. The World Health Organization started the World Health Organization’s Expanded Program on Immunization in 1974 to strengthen the fight against childhood tuberculosis in developing countries [3]. The incidence of tuberculosis is relatively high in South Korea, which implemented the National Tuberculosis Program in 1962 [1,4]. A compulsory childhood immunization program has been implemented, which reportedly vaccinates approximately 95–99% of children with BCG [1].

BCG vaccination is considered safe, but complications such as localized ulceration, regional lymphadenitis, osteomyelitis, eczema vaccinatum, hypertrophic scars and keloid formation, and disseminated infection can occur [3,5]. Some BCG complications can occur 6–9 months after vaccination. Suppurative lymphadenitis is the most common and is defined as the development of ipsilateral regional lymph node (LN) enlargement after BCG vaccination, usually occurring from 3 to 28 weeks after vaccination [5,6,7]. The rate of regional suppurative lymphadenitis ranges from about 0.1 to 38 per 1000 [5]. BCG lymphadenitis may undergo spontaneous regression or become progressively enlarged [3]. Several treatment methods, such as surgical excision and antibiotics, have been administered, without any definitive proof of efficacy; therefore, there is still no consensus on the most appropriate treatment for BCG lymphadenitis [3].

In the current study, patient characteristics and clinical courses of BCG lymphadenitis were investigated.

## 2. Materials and Methods

The present study included patients who visited one institute with a diagnosis of BCG lymphadenitis between January 2012 and June 2017. The data were acquired retrospectively via medical chart review. They included sex, age at first visit, vaccination route, time from BCG injection to symptom onset, lesion location, LN size determined via physical examination and imaging, imaging modalities used, treatment methods, departments involved in treatment, lesion status (clear or remaining) at last visit and at the time of a current phone interview, and follow-up duration. In case of patients who diagnosed with chronic granulomatous disease were excluded.

The diagnostic criteria included a history of BCG vaccination on the symptomatic side, absence of tenderness and raised temperature in the region of swelling, absence of fever and constitutional symptoms, and isolated axillary (or supraclavicular/cervical) LN enlargement. Treatment methods were divided into four groups; observation, antibiotics, incision and drainage or needle aspiration (I&D/NA), and surgical excision, and included treatments performed previously at other hospitals. Current status and recurrence were evaluated via telephone interviews. The follow-up period was from the first to the last visit to the out-patient clinic, including visits occurring previously at other hospitals. “Clear of lesion” was defined as no residual lesion on medical record.

Chi-square tests, *t*-tests, and logistic regression analysis were used to compare groups. Frequencies and percentages were analyzed for categorical variables, and means, standard deviations, medians, and ranges were analyzed for continuous variables. All tests were considered statistically significant at *p* < 0.05. All statistical analyses were performed using SPSS version 22 (IBM Corporation, Sommers, NY, USA). Institutional review board approval was obtained before the initiation of the study (approval number 1807-007-955).

## 3. Results

A total of 171 patients were included in the study, of which 108 (63.2%) were male and 63 (36.8%) were female. The median age at the first visit was 5.5 months (range 0.9–83.7 months). The majority were vaccinated percutaneously (111; 64.9%), and the remainder were vaccinated intradermally (55; 32.2%). In Korea, Danish 1331 strain is used for percutaneous vaccination and Tokyo 172 strain is used for intradermal administration. The most common location of BCG lymphadenitis was the axilla (81.3%). The respective mean LN sizes determined via physical examination and imaging were 1.63 ± 0.87 cm and 1.87 ± 0.71 cm. The mean interval between BCG vaccination and symptom onset was 60 days (range of 14–710 days). The recurrent symptom was evident in 26 patients (15.2%). The treatment methods used, including those applied previously at other hospitals, were observation (57.9%), I&D/NA (27.5%), surgical excision (11.7%), and antibiotics (2.9%). The median follow-up period was 47 days (range 1–1245 days) (Table 1).

Patient demographics and treatment methods were analyzed. LN sizes determined via physical examination were significantly larger in the I&D/NA group than in the antibiotics group (2.2 ± 0.9 cm vs. 1.0 ± 0.0 cm, *p* = 0.032). LN sizes determined via imaging were significantly larger in the I&D/NA group than in the observation group (2.7 ± 0.7 cm vs. 1.9 ± 0.6 cm, *p* < 0.001). There were no significant differences in age at first visit, sex, vaccination route, LN location, and interval between vaccination and symptom onset, presence of the recurrent symptom, or follow-up period between the treatment groups (Table 2).

Factors associated with lesion reduction were compared. The surgical excision group exhibited the smallest percentage of remaining lesions at the last visit, and that percentage was significantly lower than that in the observation group (RR 17.206, CI 3.768–78.561, *p* < 0.001). There were no significant differences in remaining lesions between any groups at the “current interview” time point (Table 3).

**Figure 1:**
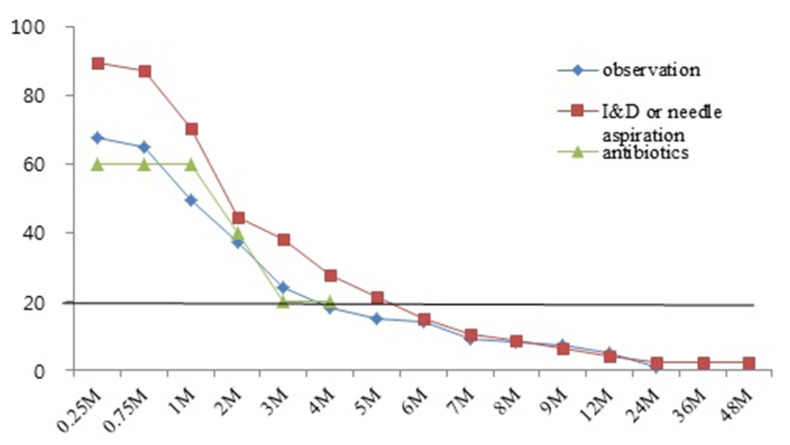
Percentages of remaining lesions in the different treatment groups (Observation, I&D or needle aspiration, antibiotics).

**Figure 2:**
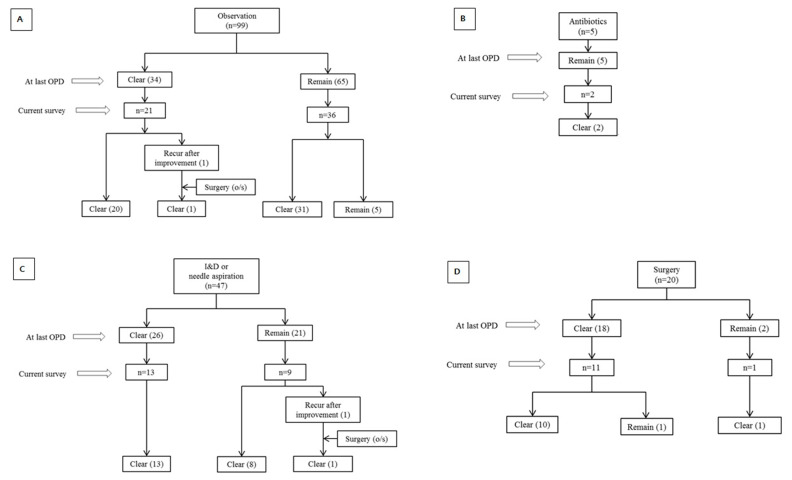
Clinical courses in the different treatment groups: (**A**) observation group; (**B**) antibiotics group; (**C**) incision and drainage or needle aspiration group; (**D**) surgery group. OPD, outpatient clinic; o/s, outside.

The times taken for lesions to reduce to < 20% were approximately 3 months in the antibiotics group, 4 months in the observation group, and 5 months in the I&D/NA group (Figure 1). One patient in the observation group and one patient in the I&D/NA group experienced recurrence after improvement, and underwent surgery at other hospitals. Five patients in the observation group and one patient in the surgery group still had remaining lesions at the current interview timepoint (Figure 2).

## 4. Discussion

Several studies have investigated BCG lymphadenitis, and its incidence has varied widely. In some studies, it was as high as 4%, whereas Demmergues et al. [7] reported that while 17.8% of children who had been BCG vaccinated exhibited adverse events, lymphadenitis only occurred in 0.1%. In Korea, Kim et al. reported incidence of lymphadenitis after BCG vaccination was 0.20% in Tokyo strain intradermal and 0.69% in Danish percutaneous administration [8]. 

There is still debate on the appropriate treatment for BCG lymphadenitis. Venkataraman et al. [9] reported that 46.2% of BCG lymphadenitis patients were managed without any treatment, 20.5% were treated with medication, 30.8% were aspirated, and 2.6% underwent surgery. One report suggests that surgery is necessary due to the repeated aspiration and inflammation that can occur when the size of the LN is 3 cm [5]. Engelis et al. [10] suggested that the indications for surgical treatment based on 194 patients who underwent BCG vaccination are LN destruction with overlying skin involvement. In that study 11.7% of patients underwent surgical treatment, whereas the rest were managed conventionally. In the present study, we identified that 57.9% of patients were observed at the first visit, but still, 39.2% of patients needed invasive procedures such as I&D and excisional surgery. If patients initially visited pediatric surgery department for BCG lymphadenitis, we discussed with pediatric immunology and infectious diseases specialists for possibility of immunodeficiency disease. Then, we decided the treatment plan. 

In 2014, Daei Parizi et al. [6] reported that 23.7% of BCG lymphadenitis patients exhibited spontaneous regression after 6 months, and that an important factor influencing their clinical course was LN size. In that study, it took 3–5 months for lesions to decrease to < 20%. Goraya et al. [3] divided BCG lymphadenitis into non-suppurative and suppurative forms. The non-suppurative form is known as simple BCG lymphadenitis and usually improves spontaneously without sequelae, but in some cases, it progresses to a suppurative form. The suppurative form accounts for 30%–80% of BCG lymphadenitis and usually develops suddenly within 2–4 months of BCG vaccination. Bolursaz et al. [11] stated that the use of anti-tuberculosis medication cannot decrease the rate of progression of non-suppurative lymphadenitis to the suppurative form, and that it drained naturally or was sinus-forming, taking months to heal. Rapid progression of BCG lymphadenitis is generally accompanied by suppuration, of which secondary infections are an associated complication [3]. In the current study 26% of patients exhibited recurrence, but it did not influence the resolution of lesions. Among the different treatment groups the highest rate of recurrence was 38.3% in the I&D/NA group, but the differences between groups were not statistically significant. There was also no statistically significant effect of recurrent symptom on the resolution state at the time of the last visit or at the current interview timepoint.

In 2015, Elsidig et al. [12] published guidelines for BCG vaccine-related lymphadenitis in children. According to those guidelines, in non-suppurative lymphadenitis only close observation and follow-up are required unless the node remains persistently enlarged for more than 6 to 9 months with a size of ≥ 3 cm. In suppurative lymphadenitis, needle aspiration is recommended rather than incision and drainage, and surgical excision is considered upon failed aspiration. In the present study, in the I&D/NA group the LNs were large as determined by both physical examination and imaging. Accompanying recurrence was highly prevalent, and I&D/NA was usually performed immediately in the outpatient department. 

At the last follow-up visit, the surgical treatment group exhibited significantly more lesion resolution than the observation group. This finding suggests that surgical excision is the most reliable method for the removal of lesions. Notably, however, there were no significant differences in resolution between the treatment groups at the current interview timepoint, i.e., when a telephone interview was conducted after a median of 45 months. There was a significant difference in the follow-up period from the last visit to the current interview timepoint between clear patients and remaining patients, so further long-term evaluation of the six patients who still had lesions was necessary.

The current study had some limitations. It was a retrospective study that relied solely on medical records. We did not include patients who suffer from inborn errors of immunity, such as chronic granulomatous disease. The treatment strategies utilized were also heterogeneous, and there were patients who had visited and been managed at other hospitals previously. There were also several departments (pediatrics, pediatric surgery, emergency medicine, and others) involved in the treatment of BCG lymphadenitis at the current hospital, and a lack of a uniform guideline for the treatment of BCG lymphadenitis during the period of the study. There were generally two or three medical members of staff from any one department involved in patient treatment, so it is not possible to rule out heterogeneity within treatment guidelines and records. Notably, the rate of participation in the telephone interview (i.e., the “current interview timepoint”) was low, and data thus derived were limited in that physical or radiological evaluation of lesions could not be conducted. Lastly, there was a lack of information on the clinical course of patients who were lost to follow-up, although many were expected to have undergone further management at other hospitals.

## 5. Conclusions

In the 171 patients in the current study, LN size was the largest in the I&D/NA group. Surgical excision was the most effective treatment at the time of the last follow-up visit, but there were no differences in long-term follow-up results between the treatment groups. Lesions had generally reduced to <20% by 4 months in the observation group, 3 months in the antibiotics group, and 5 months in the I&D/NA group. Future randomized controlled studies aimed at identifying optimal treatments for BCG lymphadenitis are necessary for the development of a unified treatment guideline. Further evaluation of long-term clinical courses would also be informative to this end.

## Figures and Tables

**Table 1:** Demographics.

	*n* = 171 (%)
Sex (M:F)	108 (63.2):63 (36.8)
Age at first visit (day, median, range)	166 (26–2511)
≤6 months	100 (58.5)
7–12 months	42 (24.6)
13–18 months	9 (5.3)
>18 months	20 (11.7)
Current age (year, median, range)	4 (0.8–15.6)
Method of BCG vaccine administration	
Percutaneous	111 (64.9)
Intradermal	55 (32.2)
Location	
Axilla	139 (81.3)
Supraclavicular	18 (10.5)
Cervical	14 (8.2)
Size of LN (cm, mean ± SD)	
P/E	1.63 ± 0.87
Imaging study	1.87 ± 0.71
Interval between BCG injection and onset of symptom (day, median, range)	0 (14–710)
recurrent symptom	6 (15.2)
Previous treatment	
Antibiotic	5 (2.9)
I & D	5 (2.9)
Surgery	1 (0.6)
Imaging study	46 (26.9)
Only USG	45 (26.3)
Only CT	1 (0.6)
USG+CT	1 (0.6)
Treatment (including previous treatment)	
Observation	99 (57.9)
I&D or needle aspiration	47 (27.5)
Surgery	20 (11.7)
Antibiotics	5 (2.9)
Follow-up period (median, range)	
First visit ~ Last visit (day)	47 (1–1245)
Last visit ~ Current survey (month)	45 (4–186)

LN, lymph node; P/E, physical examination; SD, standard deviation; I&D, incision and drainage; USG, ultrasonography; CT, computed tomography.

**Table 2:** Demographics according to treatment.

	Observation *n* = 99 (%)	Antibiotics *n* = 5 (%)	I&D or Needle Aspiration *n* = 47 (%)	Surgery *n* = 20 (%)	*p*-Value
Age at first visit (day, mean ± SD)	320.3 ± 46.7	287.8 ± 31.9	188.5 ± 153.9	340.2 ± 359.4	-
Sex					
Male	56 (56.6)	4 (80)	36 (76.6)	12 (60)	0.329
Female	43 (43.4)	1 (20)	11 (23.4)	8 (40)	
Method of BCG administration					
Percutaneous	68 (68.7)	3 (60.0)	25 (53.2)	15 (75.5)	0.853
Intradermal	29 (29.3)	2 (40.0)	20 (43.7)	4 (20.0)	
Location of LN					
Axilla	78 (78.8)	3 (60)	41 (87.2)	17 (85)	0.405
Supraclavicular	11 (11.1)	0 (0)	4 (8.5)	3 (15)	
Cervical	10 (10.1)	2 (40)	2 (4.3)	0 (0)	
Size of LN (cm, mean ± SD)					
P/E	1.4 ± 0.8	1.0 ± 0.0	2.2 ± 0.9	1.7 ± 0.8	0.032 *
Imaging study	1.9 ± 0.6	1.7 ± 1.1	2.7 ± 0.7	1.5 ± 0.6	<0.001 ^†^
Interval between BCG injection and onset of symptom (day, mean ± SD)	93.4 ± 114.0	64.0 ± 0.0	73.4 ± 40.9	92.3 ± 73.1	-
recurrent symptom	6 (6.1)	0 (0.0)	18 (38.3)	2 (10.0)	0.182
Follow-up period (median, range)					
First visit ~ Last visit (day)	30 (1–525)	26 (1–67)	74 (1–1245)	130 (16–385)	N/A
Last visit ~ Current survey (month)	31 (4–55)	71 (70–73)	60 (23–71)	75 (20–186)	N/A

I&D, incision and drainage; SD, standard deviation; LN, lymph node; P/E, physical examination; RR, relative risk; * Size of LN: antibiotics < needle aspiration or I&D; ^†^ Size of LN: observation < needle aspiration or I&D.

**Table 3:** Factors associated with resolution of lesion.

	Clear	Remain			
	At Last Visit		Univariate	Multivariate	
	*n* = 73 (%)	*n* = 98 (%)	*p*-Value	RR	*p*-Value
Age at first visit (day, mean ± SD)	242.38 ± 29.32	335.26 ± 45.63	0.109		
Sex					
Male	47 (64.4)	61 (61.6)	0.774		
Female	26 (35.6)	37 (38.4)			
Method of BCG administration					
Percutaneous	48 (65.7)	63 (63.6)	0.962		
Intradermal	24 (32.9)	31 (31.3)			
Location of LN					
Axilla	60 (82.2)	79 (79.8)	0.461		
Supraclavicular	9 (12.3)	9 (9.1)			
Cervical	4 (5.5)	10 (10.1)			
Department					
Only Pediatrics	34 (46.6)	73 (73.7)	0.001		
Pediatrics and Pediatric surgery	17 (23.3)	13 (13.1)			
Pediatric surgery	15 (20.6)	8 (8.1)			
ENT	6 (8.2)	2 (2.1)			
Others	1 (1.4)	2 (2.1)			
Size of LN (cm, mean ± SD)					
P/E	1.62 ± 0.90	1.63 ± 0.86	0.965		
Imaging study	1.70 ± 0.70	2.05 ± 0.69	0.165		
Interval between BCG injection and onset of symptom (day, mean ± SD)	80.21 ± 49.15	91.20 ± 113.42	0.581		
recurrent symptom	12 (16.4)	14 (14.3)	0.698		
Treatment				Reference	
Observation	34 (46.6)	65 (65.7)	<0.001	RR 0.000	0.999
Antibiotics	0 (0.0)	5 (5.1)		RR 1.544 (0.760–3.138)	0.230
Needle aspiration or I&D	21 (28.8)	26 (26.3)		RR 17.206 (3.768–78.561)	<0.001
Surgery	18 (24.7)	2 (2.1)			
Follow-up period (median, range) First visit ~ Last visit (day)	37 (1–455)	32 (1–1245)	0.909		
	**At Current Survey**				
	***n* = 87 (%)**	***n* = 6 (%)**			
Age at first visit (day, mean ± SD)	255.40 ± 25.25	343.33 ± 29.25	0.418		
Sex					
Male	51 (58.6)	5 (83.3)	0.234		
Female	36 (41.4)	1 (16.7)			
Method of BCG administration					
Percutaneous	59 (67.8)	6 (100.0)	0.098		
Intradermal	28 (32.2)	0 (0.0)			
Location of LN					
Axilla	71 (81.6)	6 (100)	0.284		
Supraclavicular	11 (12.6)	0 (0.0)			
Cervical	5 (5.7)	0 (0.0)			
Department					
Only Pediatrics	56 (64.4)	5 (83.3)	0.526		
Pediatrics and Pediatric surgery	17 (19.6)	0 (0.0)			
Pediatric surgery	10 (11.5)	1 (16.7)			
ENT	3 (3.4)	0 (0.0)			
Others	1 (1.1)	0 (0.0)			
Size of LN (cm, mean ± SD)					
P/E	1.62 ± 0.91	2.13 ± 1.44	0.299		
Imaging study	1.86 ± 0.75	-	-		
Interval between BCG injection and onset of symptom (day, mean ± SD)	91.12 ± 67.38	94.00 ± 66.84	0.944		
recurrent symptom	12 (13.8)	0 (0.0)	0.332		
Treatment					
Observation	52 (59.8)	5 (83.3)	0.681		
Antibiotics	2 (0.23)	0 (0.0)			
Needle aspiration or I&D	22 (25.3)	0 (0.0)			
Surgery	11 (12.6)	1 (16.7)			
Follow-up period (median, range) Last visit ~ Current survey (month)	49 (1–145)	18 (5–59)	0.016		

I&D, incision and drainage; SD, standard deviation; LN, lymph node; P/E, physical examination; RR, relative risk; ENT, ear, nose, and throat department.

## Data Availability

Not applicable.

## References

[1] Baek S.O., Ko H.S., Han H.H. (2017). BCG vaccination-induced suppurative lymphadenitis: Four signs to pay attention to. Int. Wound J..

[2] Cuello-Garcia C.A., Perez-Gaxiola G., Jimenez Gutierrez C. (2013). Treating BCG-induced disease in children. Cochrane Database Syst. Rev..

[3] Goraya J.S., Virdi V.S. (2002). Bacille Calmette-Guerin lymphadenitis. Postgrad. Med. J..

[4] Kim J.H., Yim J.J. (2015). Achievements in and Challenges of Tuberculosis Control in South Korea. Emerg. Infect. Dis..

[5] Nazir Z., Qazi S.H. (2006). Bacillus Calmette-Guerin (BCG) lymphadenitis-changing trends and management. J. Ayub Med. Coll. Abbottabad..

[6] Daei Parizi M., Kardoust Parizi A., Izadipour S. (2014). Evaluating clinical course of BCG lymphadenitis and factors affect on it during a 5-year period in Kerman, Iran. J. Trop. Pediatr..

[7] Dommergues M., de La Rocque F., Guy C., Lécuyer A., Jacquet A., Guérin N., Fagot J., Boucherat M., D’Athis P., Cohen R. (2009). Local and regional adverse reactions to BCG-SSI® vaccination: A 12-month cohort follow-up study. Vaccine.

[8] Kim J., Lee K., Kim J.H., Kim S.J., Lee S.Y., Lee H.J., Cho K.S., Kwon Y.J., Lee B.C., Jo S.M. (2016). The incidence rate of lymphadenitis after Bacille Calmette-Guerin vaccination. Pediatr. Infect. Vaccine.

[9] Venkataraman A., Yusuff M., Liebeschuetz S., Riddell A., Prendergast A.J. (2015). Management and outcome of Bacille Calmette-Guerin vaccine adverse reactions. Vaccine.

[10] Engelis A., Kakar M., Meiksans R., Petersons A. (2016). BCG-SSI(®) vaccine-associated lymphadenitis: Incidence and management. Medicina.

[11] Bolursaz M.R., Lotfian F., Velayati A.A. (2017). Bacillus Calmette-Guerin vaccine complications in Iranian children at a university hospital. Allergol. Immunopathol..

[12] Nagi E., Dayel A., Mohammed A., Mohammed A., Sami A., Suliman A., Ibrahim Bin H., Mohammad A., Fahad A., Abdularahman A. (2015). Bacillus Calmette–Guérin vaccine related lymphadenitis in children: Management guidelines endorsed by the Saudi Pediatric Infectious Diseases Society (SPIDS). Int. J. Pediatr. Adolesc. Med..

